# 17,β‐estradiol inhibits hepatitis C virus mainly by interference with the release phase of its life cycle

**DOI:** 10.1111/liv.13303

**Published:** 2016-11-25

**Authors:** Andrea Magri, Matteo N. Barbaglia, Chiara Z. Foglia, Elisa Boccato, Michela E. Burlone, Sarah Cole, Paola Giarda, Elena Grossini, Arvind H. Patel, Rosalba Minisini, Mario Pirisi

**Affiliations:** ^1^ Department of Translational MedicineUniversità del Piemonte OrientaleNovaraItaly; ^2^ MRC‐University of Glasgow Centre for Virus ResearchGlasgowUK; ^3^ CRRF Mons. Luigi NovareseMoncrivelloVercelliItaly

**Keywords:** 17β‐estradiol, antivirals, hepatitis C virus, oestrogen

## Abstract

**Background & Aims:**

Oestrogen and oestrogen‐mediated signalling protect from hepatitis C virus through incompletely understood mechanisms. We aimed to ascertain which phase(s) of hepatitis C virus life cycle is/are affected by oestrogens.

**Methods:**

Huh7 cells infected with the JFH1 virus (genotype 2a) were exposed to dehydroepiandrosterone, testosterone, progesterone and 17β‐estradiol (tested with/without its receptor antagonist fulvestrant). Dose–response curves were established to calculate half maximal inhibitory concentration values. To dissect how 17β‐estradiol interferes with phases of hepatitis C virus life cycle, its effects were measured on the hepatitis C virus pseudo‐particle system (viral entry), the subgenomic replicon N17/JFH1 and the replicon cell line Huh7‐J17 (viral replication). Finally, in a dual‐step infection model, infectious supernatants, collected from infected cells exposed to hormones, were used to infect naïve cells.

**Results:**

Progesterone and testosterone showed no inhibitory effect on hepatitis C virus; dehydroepiandrosterone was only mildly inhibitory. In contrast, 17β‐estradiol inhibited infection by 64%‐67% (IC_50_ values 140‐160 nmol/L). Fulvestrant reverted the inhibition by 17β‐estradiol in a dose‐dependent manner. 17β‐estradiol exerted only a slight inhibition (<20%) on hepatitis C virus pseudo‐particles, and had no effect on cells either transiently or stably (Huh7‐J17 cells) expressing the N17/JFH1 replicon. In the dual‐step infection model, a significant half maximal inhibitory concentration decline occurred between primary (134 nmol/L) and secondary (100 nmol/L) infections (*P*=.02), with extracellular hepatitis C virus RNA and infectivity being reduced to a higher degree in comparison to its intracellular counterpart.

**Conclusions:**

17β‐estradiol inhibits hepatitis C virus acting through its intracellular receptors, mainly interfering with late phases (assembly/release) of the hepatitis C virus life cycle.

**Abbreviations**
BAF: bafilomycin A
BSA: bovine serum albumin
cDNA: complementary deoxyribonucleic acid
CMA: 2′‐C‐methyladenosine
DAA: direct antiviral agents
DHEA‐S: dehydroepiandrosterone sulphate
DMEM: Dulbecco's modified Eagle's medium
DMSO: dimethyl sulphoxide
E_2_: 17β‐estradiol
ERαβ: oestrogen intracellular receptor
FFU: focus forming units
GPER: G protein‐coupled oestrogen receptor 1
HCV: Hepatitis C Virus
HCVcc: Hepatitis C Virus cell‐cultured
HCVpp: HCV pseudoparticles
IC_50_: half maximal inhibitory concentration
IgG: Immunoglobulin G
JAK/STAT: Janus kinase/signal transducer and activator of transcription
MOI: multiplicity of infection
MTP: Microsomal triglyceride transfer protein
MTT: methylthiazolyldiphenyl‐tetrazolium bromide
MxA: human myxovirus resistance protein 1
NTR: non‐translated region
qPCR: quantitative polymerase chain reaction
RLU: relative light units
SREBPs: Sterol regulatory element‐binding proteins
TLR‐7: Toll‐like receptor 7

1

**Key points**

- Among sex hormones, only 17,β‐estradiol exhibits antiviral properties against HCV in two different models of infection.
- The oestrogen‐related antiviral effect is abrogated in a dose‐dependent manner by the oestrogen‐receptor modulator, fulvestrant, confirming that the antiviral action of 17,β‐estradiol is indirect.
- Oestrogen stimulation partially reduces viral entry, but has no effect on viral RNA replication.
- 17,β‐estradiol blocks HCV infection also by interfering with a late phase of viral life cycle, (viral assembly/release), reducing the number of viral particles released by infected cells.

## 1 Introduction

In contrast with the authentic revolution we are facing in the field of antiviral therapy of hepatitis C, several long‐standing issues concerning the natural history of HCV infection remain incompletely understood. Specifically, it has always been puzzling why gender affects so deeply the course of hepatitis C. It has been consistently shown along the years that the ability to spontaneously clear HCV infection is greater in women than in men.^1^, ^2^, ^3^, ^4^, ^5^ Moreover, during early chronic infection (1‐year post‐infection), HCV RNA levels are higher in men than in women,^6^ while cirrhotic progression rarely occurs in pre‐menopausal women.^7^ Based on these observations, it has been proposed that oestrogen and oestrogen‐mediated signalling may play a role as protective factors, reducing the disease progression or increasing the chance of clearing the virus.^8^ The mechanisms by which this protection occurs are unknown; the prevailing hypothesis advocates that sex hormones bind to specific receptors expressed in immune cells, thereby influencing adaptive and, most importantly, innate immune responses.^9^, ^10^ However, oestrogens are not key players in the major mechanism of innate antiviral response, the JAK/STAT pathway,^11^ and in other settings, the major impact of sex steroids is on the target tissue, not on immune modulation.^12^ Thus, it is not inconceivable that oestrogens may determine a suboptimal environment for viral replication by completely different mechanisms. This possibility is nicely exemplified by a recent study demonstrating that oestrogens favour the cleavage of the tight junction protein occludin, one of the proteins HCV uses to gain access to the hepatocyte.^13^

Better understanding of the mechanisms by which factors modulating the natural history of HCV infection act remains a compelling issue in the DAA era, since new anti‐HCV drugs are so expensive that many countries restrict their use to patients with significant fibrosis progression. With the present in vitro study, we aimed to better define the antiviral properties of sex hormones on HCV infection, and in particular to ascertain which phase(s) of HCV life cycle is/are affected by oestrogens.

## 2 Methods

### 2.1 Hormones and chemicals

17,β‐estradiol (E2), testosterone, progesterone, dehydroepiandrosterone‐sulphate (DHEA‐S), Fulvestrant, the entry inhibitor Bafilomycin A1 (BAF)^14^ and the replication inhibitor 2′‐C‐methyladenosine (CMA)^15^ were obtained from Sigma‐Aldrich (Milan, Italy), dissolved as indicated and stored at −20°C.

### 2.2 Cell cultures

Human hepatoma Huh7 cells,^16^ Human epithelial kidney cells 293T and Huh7‐J17 cells were grown as previously reported.^17^

### 2.3 Plasmids and in vitro transcription

The plasmid pJFH1, containing the full‐length genomic cDNA sequence of the HCV genotype 2a strain and the N17/JFH1^17^, ^18^ plasmid were linearized using *XbaI* enzyme (New England Biolabs) and then treated with Mung Bean Nuclease (NEB) prior purification. Linearized plasmids were used as a template to generate in vitro transcribed RNA using MEGAscript T7 (Life Technologies, Milan, Italy). The 10 μg of RNA were electroporated into Huh7 cells as previously described.^19^

### 2.4 Antiviral evaluation on HCVcc

**Figure 1:**
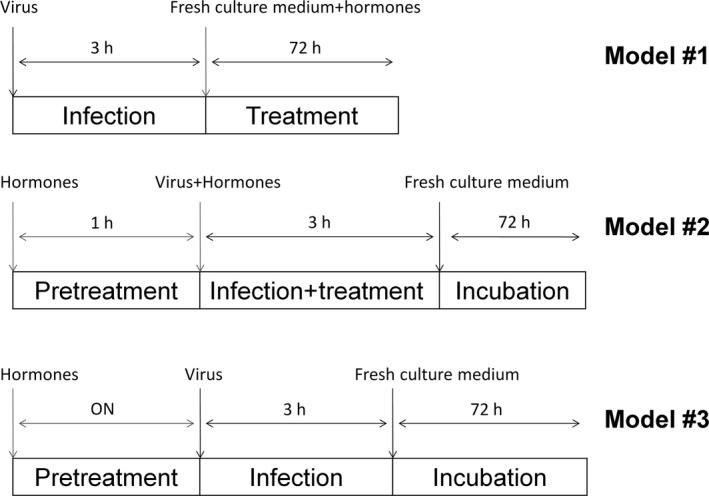
Schematic representation of infection models. In model #1 cells were infected and then treated with E2 for 72 h. In model #2 cells were pretreated for 1 h and then exposed with E2 during the first 3 h of infection. In model #3 cells were pretreated overnight and then infected in absence of E2

Huh7 cells were infected with JFH1 virus at a multiplicity of infection (MOI) of 0.1. The infection was performed as follow using three different models to derive hormone dose–response scales, as shown in Figure 1. In model #1, Huh7 cells were infected in the absence of hormones: after 3 hours the inoculum was replaced with fresh complete medium containing various hormone concentrations. In model #2, 1 hour before infection the cells were pre‐treated with each hormone. Subsequently the cells were infected in the presence of the compounds and incubated at 37°C. After the 3 hours the viral inoculum was removed, cells washed and re‐fed with complete medium and incubated at 37°C for 72 hours. In model #3, Huh7 cells were pre‐treated overnight with hormones, then washed and infected in the absence of drugs.

Viral inhibition was evaluated counting the focus forming units (FFU) after immune staining on fixed cells; results were normalized to DMSO‐treated cells (drug vehicle control).

### 2.5 Combination treatment

Huh7 cells were exposed to 17,β‐estradiol at the concentration of 200 nmol/L, plus different concentrations of DHEA‐S or testosterone as reported in Table 1; in addition, the infections were performed according to the models #2 and #3.

**Table 1:** Hormone concentrations tested in the experiments

	17,β‐estradiol, nmol/L	Fulvestrant, nmol/L	Progesterone, μmol/L	Testosterone, μmol/L	DHEA‐S
Concentrations	400	400	25	10	50 mmol/L
	200	200	12.5	5	5 mmol/L
	100	100	6.25	2.5	500 μmol/L
	50	50	3.125	1.25	50 μmol/L
	25	25	1.6	0.625	5 μmol/L
	12.5	12.5	0.8	0.3125	500 nmol/L
	6.25	6.25		0.1562	50 nmol/L

### 2.6 Dual step infection

Briefly, Huh7 cells were infected and treated with different concentrations of 17,β‐estradiol following the models #2 and #3 described above. After 3 days, cells were fixed and stained to obtain data from primary infection. The supernatants, collected from infected cells, were used to perform a secondary infection on naïve Huh7, without any treatment. After other 3 days, the antiviral effect on secondary infection was visualized as described below.

### 2.7 Immunoperoxidase staining

Infected cells were fixed in 4% paraformaldehyde. After permeabilization with Triton X‐100 0.5%, treatment with H_2_O_2_ 0.3% and saturation with BSA 3%, cells were visualized with AEC Staining Kit (Sigma Aldrich) using as primary antibody a serum of HCV positive patient, and as secondary antibody a rabbit anti‐human IgG labelled with peroxidase (Dako).

### 2.8 RNA inhibition

Huh7 cells were treated with 17,β‐estradiol at the concentration of 200 nmol/L and infected following models #2 and #3 as described above. After 72 hours intracellular RNA was purified using TRI‐reagent (Sigma‐Aldrich), while RNA from the released viral particles was extracted using PureLink Viral RNA kit (Life Technologies). Reverse transcription was performed with random primers using the same volume (10 μL) for RNAs extracted from supernatant and 1 μg for intracellular RNAs. Viral quantification of RNA was determined by RT‐qPCR using the Fast Sybr Master Mix (Life Technologies) with specific primers for the HCV 5′ NTR: 5′‐TCCCGGGAGAGCCATAGTG‐3′ (sense) 5′‐TCCAAGAAAGGACCCAGTC‐3′ (antisense) as described previously.^20^ The copy number of viral RNA was obtained by absolute quantification using a linear regression on serial dilutions of the pJFH1 plasmid at known concentrations.

### 2.9 Infectivity inhibition and specific infectivity

Huh7 cells were treated with 17,β‐estradiol as described above for RNA inhibition. After 72 hours supernatants were collected and titrated in a 96‐well plate following five‐fold dilutions or quantitated by RT‐qPCR as described above. Infected cells were washed, lysed and titrated as previously reported.^21^, ^22^ Specific infectivity was calculated as the ratio between the viral titre, expressed as FFU/mL and the number of RNA copies.

### 2.10 HCV subgenomic replicon

Huh7 cells were electroporated with N17/JFH1 RNA, seeded in the presence of different concentrations of 17,β‐estradiol or CMA at the concentration of 1 μmol/L and incubated for 24, 48 or 72 hours before measuring luciferase activity as previously reported.^17^ The replicon cell line Huh7‐J17, stably expressing viral RNA, was generated as previously described.^17^ These cells were cultured for 20 days in presence of 17,β‐estradiol at the concentration of 400 nmol/L, CMA at the concentration of 1 μmol/L or DMSO. Results were collected after 1, 2, 4, 7, 10, 14 and 20 days as luciferase readings using Bright‐Glo (Promega, Milan, Italy).

### 2.11 HCV pseudoparticles

HCV pseudoparticles (HCVpp) were generated as previously reported.^23^ To evaluate viral inhibition, Huh7 cells were pre‐treated for 1 hour with 17,β‐estradiol or BAF at the concentration of 10 nmol/L and then infected for 3 hours with HCVpp at a MOI of 0.1 according to model #2 described above. After 72 hours cells lysed and luciferase readings taken. Results were normalized to DMSO‐treated cells.

### 2.12 Statistical analysis

Statistical analysis of data was performed using the software package Stata Rel. 13.1 (StataCorp, College Station, TX, USA). The experiments were performed in triplicate and repeated at least thrice. Intracellular vs extracellular HCV RNA levels were compared by Wilcoxon ranksum tests. FFU counts observed after primary vs secondary infection at different 17,β‐estradiol concentrations were evaluated by analysis of variance with repeated measures to reject the null hypothesis that the means were the same across the groups being compared; post‐hoc pairwise multiple comparison tests were performed by the Bonferroni's method. The level of statistical significance chosen was .05, and was two‐tailed.

## 3 Results

### 3.1 Antiviral properties of sex hormones in relationship with the timing of infection and the exposure to hormones

**Figure 2:**
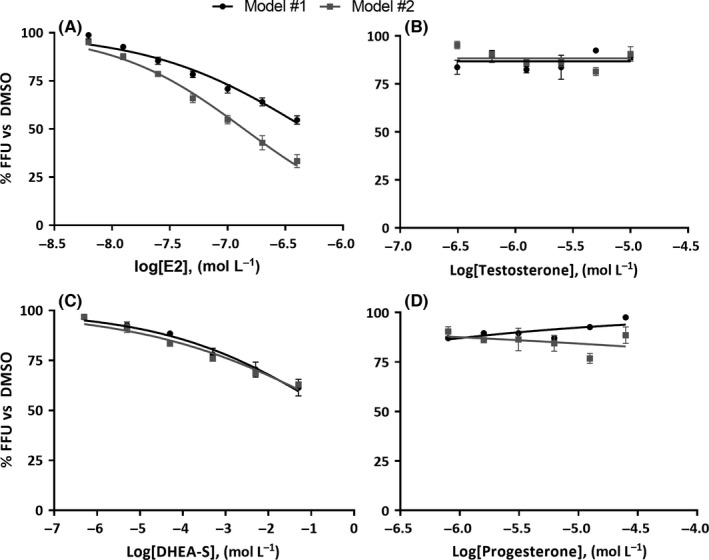
Effect of different hormones on HCVcc infection. Huh7 cells were infected in the presence of different concentration of hormones or DMSO as describe above. Results represent the percentage of focus forming units (FFU) normalized to dimethyl sulphoxide (DMSO). Results are presented both in model #1 (in which infection precedes exposure to hormone; black dots and line) and #2 (in which cells are preconditioned for 1 h and infected in the presence of hormones; grey dots and line). Panel (A), 17,β‐estradiol; panel (B), testosterone; panel (C), dehydroepiandrosterone sulphate (DHEA‐S); panel (D), progesterone

The sex hormones 17,β‐estradiol, progesterone, testosterone and DHEA‐S were tested in the three different models of infection, as described in 2, using dose–response scales covering the physiological ranges listed in Table 1 and Table S1. All the hormones were non‐cytotoxic when tested by the MTT assay (data not shown). In model #1, where infection precedes exposure to hormones, both 17,β‐estradiol and DHEA‐S showed a partial inhibition (30/40%), without reaching the IC_50_, while no effects were observed from testosterone and progesterone (Figure 2).

Data collected from model #2, where cells are preconditioned for a short time and infected in the presence of hormones, showed that 17,β‐estradiol was able to impair viral replication up to 70%, with an IC_50_ of 137 nmol/L. DHEA‐S also exhibited a moderate effect, comparable to that observed with model #1, without reaching the IC_50_. Once again, testosterone and progesterone were not able to induce a measurable antiviral response (Figure 2).

**Figure 3:**
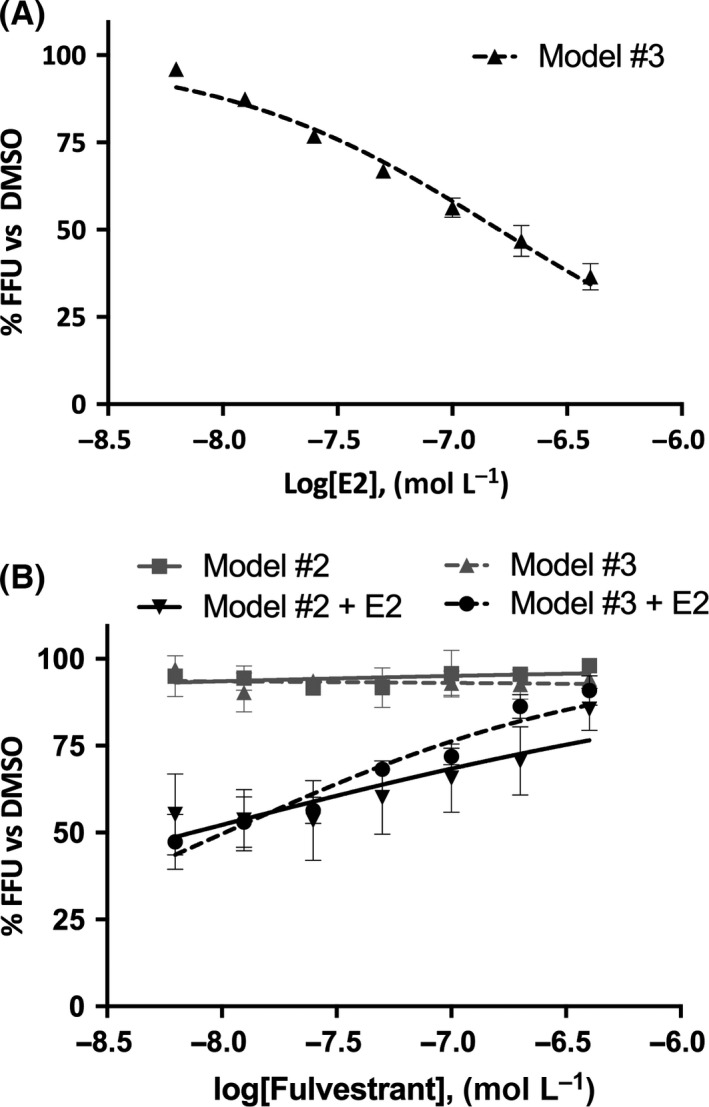
17,β‐estradiol antiviral effect is indirect and exerted through its pathway. Results show percentage of focus forming units (FFU) normalized to dimethyl sulphoxide (DMSO) at increasing 17,β‐estradiol concentrations. Panel (A), Huh7 cells were pretreated with 17,β‐estradiol and then infected, following Model #3 (overnight exposure to hormone precedes infection); panel (B), Huh7 cells were exposed to 17,β‐estradiol+fulvestrant (black symbols and line) or fulvestrant (grey symbols and line) in Model #2 (continuous line) and #3 (dashed line)

Based on these results, in model #3, where cells are preconditioned for a longer time with the hormone then left to grow in its absence after the infection, we focused our experiments on 17,β‐estradiol. Noteworthy, 17,β‐estradiol exhibited an antiviral effect up to 70%, showing the same inhibition profile observed on model #2 (Figure 3A) with an IC_50_ of 160 nmol/L.

**Figure 4:**
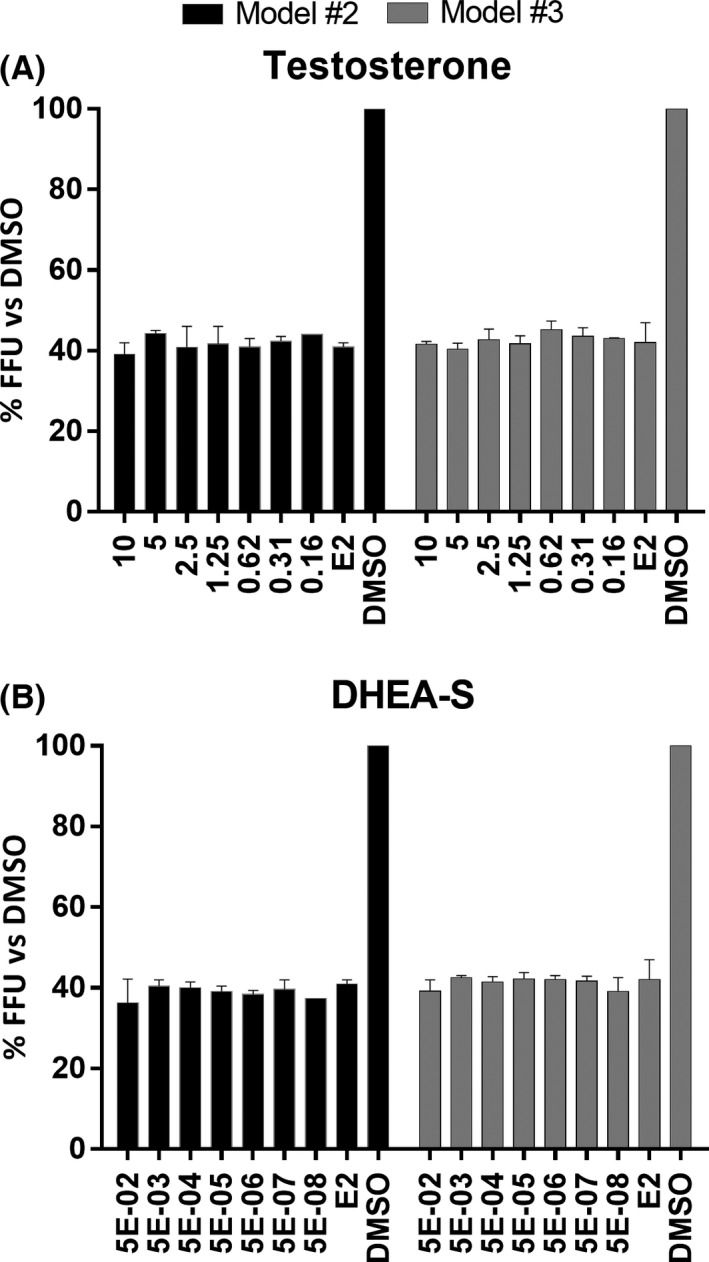
Antiviral effect of 17,β‐estradiol in combination stimulation. Huh7 cells were treated with E2 (200 nmol/L) in addition to progesterone or testosterone dose–response scales. Results represent percentage of focus forming units (FFU) normalized to dimethyl sulphoxide (DMSO) in cells exposed to 17,β‐estradiol 200 nmol/L and increasing concentrations of other hormones (black columns, Model #2; grey columns, Model #3). Panel (A), testosterone; panel (B), dehydroepiandrosterone sulphate

To simulate physiological conditions, in which different sexual hormones are present at the same time at different concentrations, we investigated if testosterone or DHEA‐S could increase the 17,β‐estradiol effect. Thus, Huh7 cells were treated with 17,β‐estradiol in combination with different concentrations of DHEA‐S or testosterone. Interestingly, neither testosterone nor DHEA‐S proved to be able to boost 17,β‐estradiol antiviral effects (Figure 4).

### 3.2 Role of intracellular oestrogen signalling in determining an antiviral state

To assess whether the antiviral effect of the 17,β‐estradiol was directly exerted on the virus rather than being dependent on intracellular signalling pathways, we investigated the role of the oestrogen intracellular receptor (ERαβ) in the antiviral response using a selective αβ‐oestrogen receptor degrader, Fulvestrant.^24^ To this purpose, Huh7 cells were treated with 200 nmol/L 17,β‐estradiol in combination with different concentrations of Fulvestrant. The experiment was performed according to the models #2 and #3 described above. As reported in Figure 3B, the inhibition induced by 17,β‐estradiol was completely abolished by Fulvestrant, in a dose‐dependent manner for both models tested, whereas the treatment with Fulvestrant alone had no effect.

### 3.3 Dissecting how oestrogens interfere with phases of HCV life cycle

**Figure 5:**
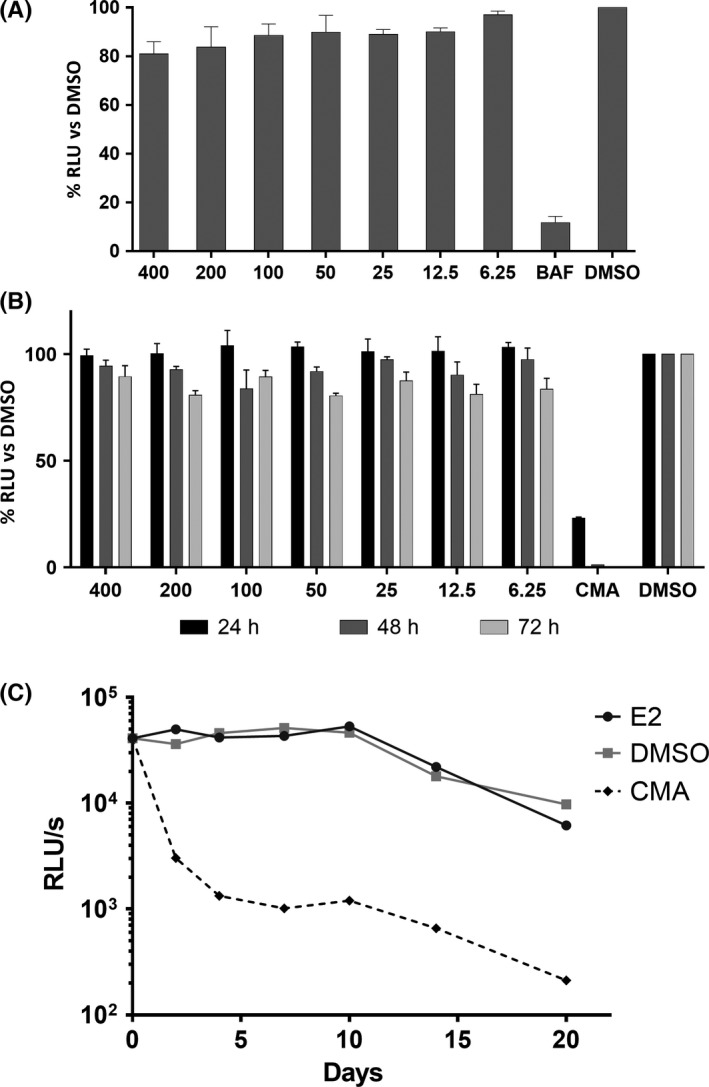
Effect of E2 on HCV entry and replication fitness. Panel (A), Huh7 cells were pretreated with E2 or DMSO and infected with HCVpp in the presence of the compounds following a dose–response scale on model #2. Antiviral effect was evaluated as infectivity of HCV pseudo‐particles, quantified as percentage of relative light units (RLU) normalized to dimethyl sulphoxide (DMSO), at increasing 17,β‐estradiol concentrations. Bafilomycin A (10 nmol/L) was used as positive control. Panel (B, C), Huh7 cells were transiently or stably transfected with N17/JFH1 replicon RNA and exposed to increasing 17,β‐estradiol concentrations or DMSO. Antiviral activity was quantified as percentage of relative light units (RLU), normalized to dimethyl sulphoxide (DMSO) and evaluated at different periods of time: 24 (black bars), 48 (dark grey bars) or 72 h (light grey bars) post‐transfection of HCV RNA (panel B); RLU per seconds obtained from a stable replicon cell line treated with 17,β‐estradiol at the concentration of 400 nmol/L for 20 days (panel C). 2′‐C‐methyladenosine (1 μmol/L) was used as positive control

We initially tested 17,β‐estradiol effect on HCV pseudoparticle system (HCVpp), which allows evaluation of the viral entry. As shown in Figure 5A, 17,β‐estradiol showed only a slight inhibition (<20%) on HCVpp, suggesting it exerts a marginal role on HCV entry.

To determine whether 17,β‐estradiol could interfere with viral replication, we used an HCV subgenomic replicon, N17/JFH1.^18^ It is a monocistronic replicon encoding non‐structural HCV proteins, structural protein Core, as reporter gene the Firefly Luciferase and the marker gene for puromycin resistance. Initially, we considered the antiviral effect on the de novo replication; results obtained on freshly transfected cells showed no significant inhibition at any concentration tested (Figure 5B). To rule out viral replication as 17,β‐estradiol target, we investigated its activity on the replicon cell line Huh7‐J17, constitutively harbouring the subgenomic RNA. No inhibition was observed across the 20 days of the experiment, as shown in Figure 5C.

**Figure 6:**
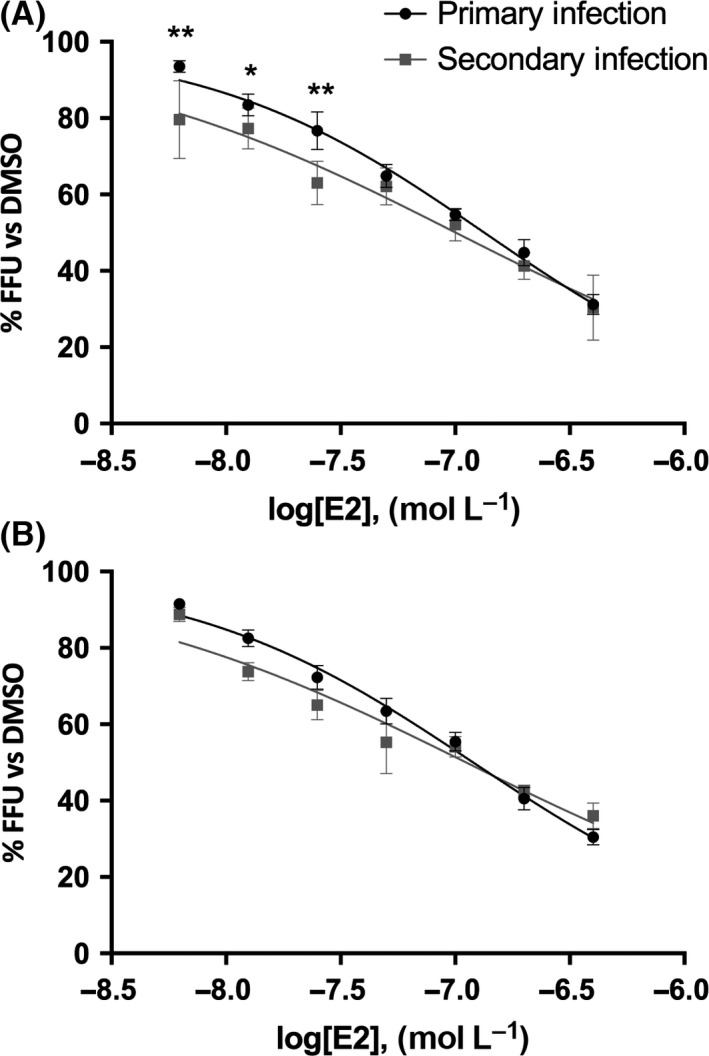
17,β‐estradiol interferes with a late phase of HCV life cycle. Huh7 cells were treated 17,β‐estradiol or DMSO and infected according to model #2 (Panel A) and #3 (Panel B). After 3 days cells were fixed and inhibition determined by immunostaining (primary infections). Supernatants were used to infect naïve cells, in absence of E2. After 3 days cells were stained (secondary infections). Results show the percentage of focus forming units (FFU) normalized to dimethyl sulphoxide (DMSO) at increasing 17,β‐estradiol concentrations during primary infection (black symbols and line). Grey symbols and line display the percentage of focus forming units (FFU) normalized to dimethyl sulphoxide (DMSO) following secondary infection. Within each group, asterisks indicate significant changes of the curve by analysis of variance for repeated measures for the trial factor: 17,β‐estradiol concentration (*P*<.001). Analysis of variance for repeated measures applies to the entire curve; however, asterisks are placed over the specific 17,β‐estradiol concentrations at which statistical significance is reached in comparison to secondary infection (Bonferroni's test for multiple comparisons). *P<.05; **P<.01

**Figure 7:**
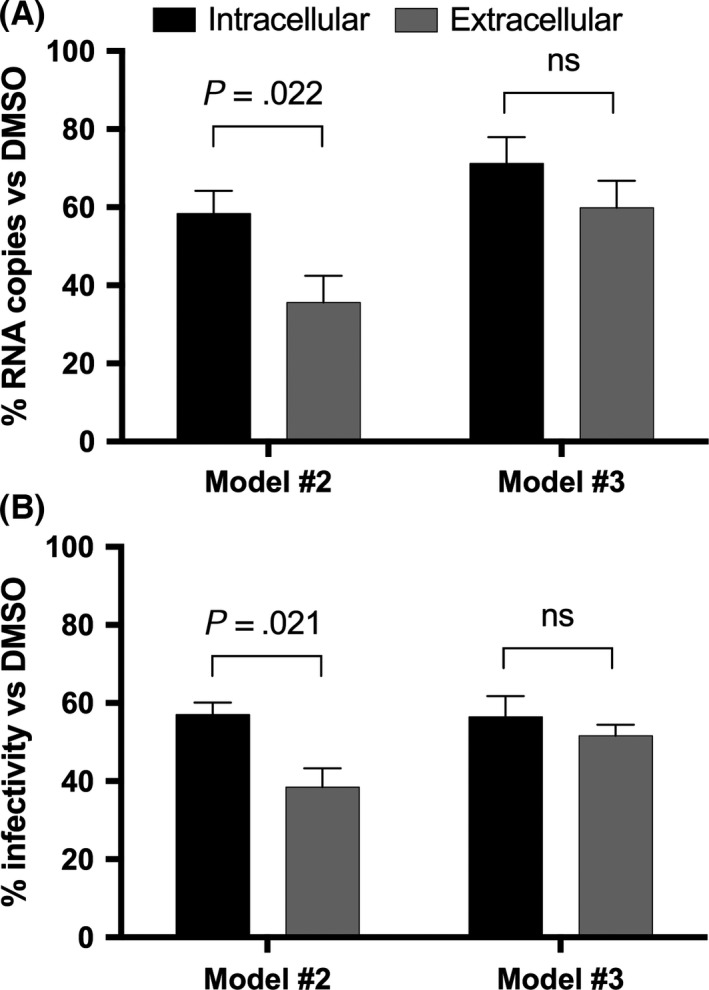
Role of 17,β‐estradiol on HCV release. Huh7 cells were pretreated with E2 or DMSO and infected according to model #2 and #3. The antiviral effect was measured at intracellular and extracellular level by RT‐qPCR (A) and infectivity (B). Panel (A), Intracellular (black columns) and extracellular (grey columns) HCV RNA (in comparison to dimethyl sulphoxide, DMSO=100%) in models #2 and #3. Panel (B), Intracellular (black columns) and extracellular (grey columns) HCV infectivity (in comparison to dimethyl sulphoxide, DMSO=100%) in models #2 and #3

Based on these results, we performed a dual‐step infection, in which infectious supernatants, collected from infected cells exposed to hormones following models #2 and #3, are used to infect naïve cells. The results obtained in the model #2 showed a significant difference between primary and secondary infection across groups, with IC_50_ values of 134 and 100 nmol/L respectively (Figure 6A). In contrast, in model #3 there was no significant variation between primary and secondary infections. The IC_50_ were 120 and 112 nmol/L respectively (Figure 6B). To validate our findings, we evaluated the inhibitory effect at extracellular and intracellular level, analysing the HCV RNA amount. As illustrated in Figure 7A, in model #2 intracellular viral RNA levels were inhibited by 40% compared to DMSO‐treated cells. Noteworthy, the viral RNA copies quantitated in the supernatant displayed a significant inhibition, up to 65% (*P*=.022). Analysing results from model #3, we observed a 30% inhibition on intracellular viral RNA, while a stronger decrease in approximately 40% was observed on extracellular RNA showing a reduced effect compared to model #2. To determine whether the effect exerted by E2 was on viral assembly or release, we investigated the intracellular and extracellular infectivity in parallel. Interestingly, in model #2 we found a 40% reduction in intracellular infectivity, and a significantly higher (62%; *P*=.021) inhibition of extracellular infectivity was observed (Figure 7B). In model #3 we detected a mitigated effect with an inhibition of intracellular and extracellular infectivity of 40% and 50% respectively. Noteworthy, these infectivity data correlate with those of viral RNA levels shown in Figure 7A. Finally, we calculated the specific infectivity, expressed as the ratio between viral titre and RNA copies; no differences were detected between control and E2 specific infectivity (Figure S1).

## 4 Discussion

In the present paper, we show that 17,β‐estradiol is able to inhibit HCV life cycle in vitro, whereas no effects were observed from exposure to DHEA‐S, testosterone or progesterone. 17,β‐estradiol acts through its intracellular receptor signalling pathway, triggering an antiviral state able to interfere with HCV assembly and/or release and partially with viral entry, but not on viral RNA replication.

With regard to the anti‐HCV effects exerted by 17,β‐estradiol, our data are in agreement with what has already been reported by Hayashida et al.,^25^ inasmuch as we both show that exposure to oestrogen inhibits the production of infectious HCV particles in vitro. However, our results diverge from those of our Hayashida et al., on the timing of exposure associated with the maximal inhibitory effect: in fact, they observed that exposure to oestrogens after viral entry led to an increased inhibition, whereas we found that exposing cells to 17,β‐estradiol before the infection clearly enhances the antiviral effect. Differences in the experimental conditions may explain this discrepancy. In the models used by Hayashida et al., viral infection was performed with a high ratio of virus to cell (multiplicity of infection, MOI), while in this study a low MOI was tested. Our choice was dictated by the following two considerations (i) low MOI are likely closer to natural conditions, and (ii) preliminary experiments by our group (data not shown) indicating that high MOI resulted in no significant antiviral activity in the model #2 and #3.

We observed no effects on HCV in vitro infection from exposure to either testosterone or progesterone. While the lack of antiviral effects of testosterone was expected, since serum testosterone levels of infected male patients correlate with an increased risk of advanced liver disease,^26^ the absence of proviral effects by these two hormones is noteworthy. Progesterone not only acts in the opposite direction of estradiol during conditions of oxidative stress,^27^ but it can also block interferon signalling pathway interfering with the expression of TLR‐7 and MxA in HCV infected people, impairing the immune response.^28^ Under natural conditions, sex hormones are simultaneously present at different concentrations in the blood stream: therefore, we tested 17,β‐estradiol in combination with testosterone or DHEA‐S showing that the antiviral effect observed by stimulation with the oestrogen alone is neither increased nor diminished by exposure to a mixture of hormones. These data are in agreement with older findings, indicating that a precursor of oestrogen and testosterone, such as DHEA‐S,^29^ cannot act synergistically with estradiol to increase its antiviral effect.

Hence, the effect on HCV is specific of oestrogens, and is mediated by its intracellular receptor. In fact, adding to the model the oestrogen receptor (αβ) antagonist fulvestrant, which impairs receptor dimerization, can revert the inhibitory effect of 17,β‐estradiol in a dose‐dependent manner. In other words, 17,β‐estradiol is not a direct antiviral agent, in full agreement with what already reported by others.^25^

Finally, we designed a series of experiments to determine which viral step(s) is/are affected by 17,β‐estradiol using surrogate infectious/replicative models. Initially, we investigated its effect on viral entry employing pseudo‐particles and obtaining only a slight to moderate inhibition. This inhibitory effect is likely to be dependent on G protein‐coupled oestrogen receptor 1 (GPER), a membrane receptor able to bind only to oestrogens and responsible for rapid effects on target cells.^30^ This hypothesis is supported by recent data showing that GPER is responsible of inhibiting HCV entry inducing a cleavage of Occludin I by metallo‐protease9 (MMP). Noteworthy, this mechanism seems to be predominant when cells reach confluence, affecting cell‐to‐cell spread through tight junctions^13^: the lower inhibitory effect under our experimental conditions could thus be explained by lower confluence conditions, affecting cell‐to‐cell spread.

As already proposed by both Hayashida et al. and Ulitzky et al.,^13^, ^25^ 17,β‐estradiol can not affect viral replication. Here, we provide additional experimental evidence consolidating this interpretation. The experiments on which the aforementioned authors based their conclusions were conducted using only HCVcc, a system that support multiple cycles of infection, making it difficult to accurately evaluate the role of viral RNA replication itself. To overcome this limitation, we electroporated cells with a subgenomic replicon and exposed them immediately to oestrogens, demonstrating that oestrogens cannot affect de novo viral replication. Moreover, we showed that stimulating replicon cells constitutively expressing subgenomic RNA up to 20 consecutive days, does not reduce viral RNA levels.

Interestingly, we observed, as already reported by Ulitzky et al.,^13^ that exposure to oestrogens leads to a lower number of foci, characterized also by a reduced size (Figure S2). On these basis, we hypothesized that 17,β‐estradiol can interfere with assembly/release of HCV infection. 17,β‐estradiol does block HCV infection in a late phase, as shown by measuring viral inhibition in exposed cells and, in parallel, viral infectivity of released particles. We observed a more profound inhibition in released particles and extracellular RNA than in intracellular RNA and infectivity, a finding confirmed by RT‐qPCR and by infectious unit titration assay. Overall, these data suggest that oestrogen can impair a late phase of HCV life cycle, mainly interfering with the release of new particles, as shown by the significant difference between intracellular and extracellular reduction (Figure 7). This observation finds further evidence showing a moderate inhibition in the intracellular infectivity. To support this hypothesis, we found no effect on specific infectivity (Figure S1), confirming that the de novo viral particles are assembled properly. It is tempting to speculate that interference with the release phase might involve the SREBP pathway, which has been shown to be activated by oestrogen.^31^ Interestingly, SREBP is able in vitro to modulate MTP,^32^ which is crucial for the correct release of infectious particles,^21^ playing a role in the regulation of the presence of ApoE on the nascent particles.^33^ In addition, the protein cholesterol‐25‐hydroxylase, known to alter the cholesterol content of membranes,^34^ it has been recently reported to affect the formation of the membranous web.^35^ Moreover, the antiviral properties could be dependent on the protective effect on lipid peroxidation on liver^36^, ^37^ resulting in an endogenous regulation of HCV life cycle, as recently reported.^38^ These hypotheses need to be tested in appropriately designed experiments; moreover, it is necessary to verify that the antiviral effects of 17,β‐estradiol are similar in all viral genotypes. Meanwhile, the safety (and possibly the benefits) of oestrogen therapy in women with chronic viral hepatitis, discouraged in the distant past because of the theoretical risk of provoking or enhancing cholestasis, finds further support in our findings.

In conclusion, our study shows that 17,β‐estradiol impairs HCV life cycle acting through its intracellular receptors and determining a massive reduction in released particles through interference with early (viral entry) and, above all, late (assembly/release) phases of the HCV life cycle.

## Conflict of interest

None.

## Supporting information

Additional Supporting Information may be found at onlinelibrary.wiley.com/doi/10.1111/liv.13303/suppinfo
