# Supplementary figures and images for: 17,β‐estradiol inhibits hepatitis C virus mainly by interference with the release phase of its life cycle
Source: Liver Int. 2016 Nov 25;37(5):669–77. doi: 10.1111/liv.13303 (PMC5448036; doi:10.1111/liv.13303)

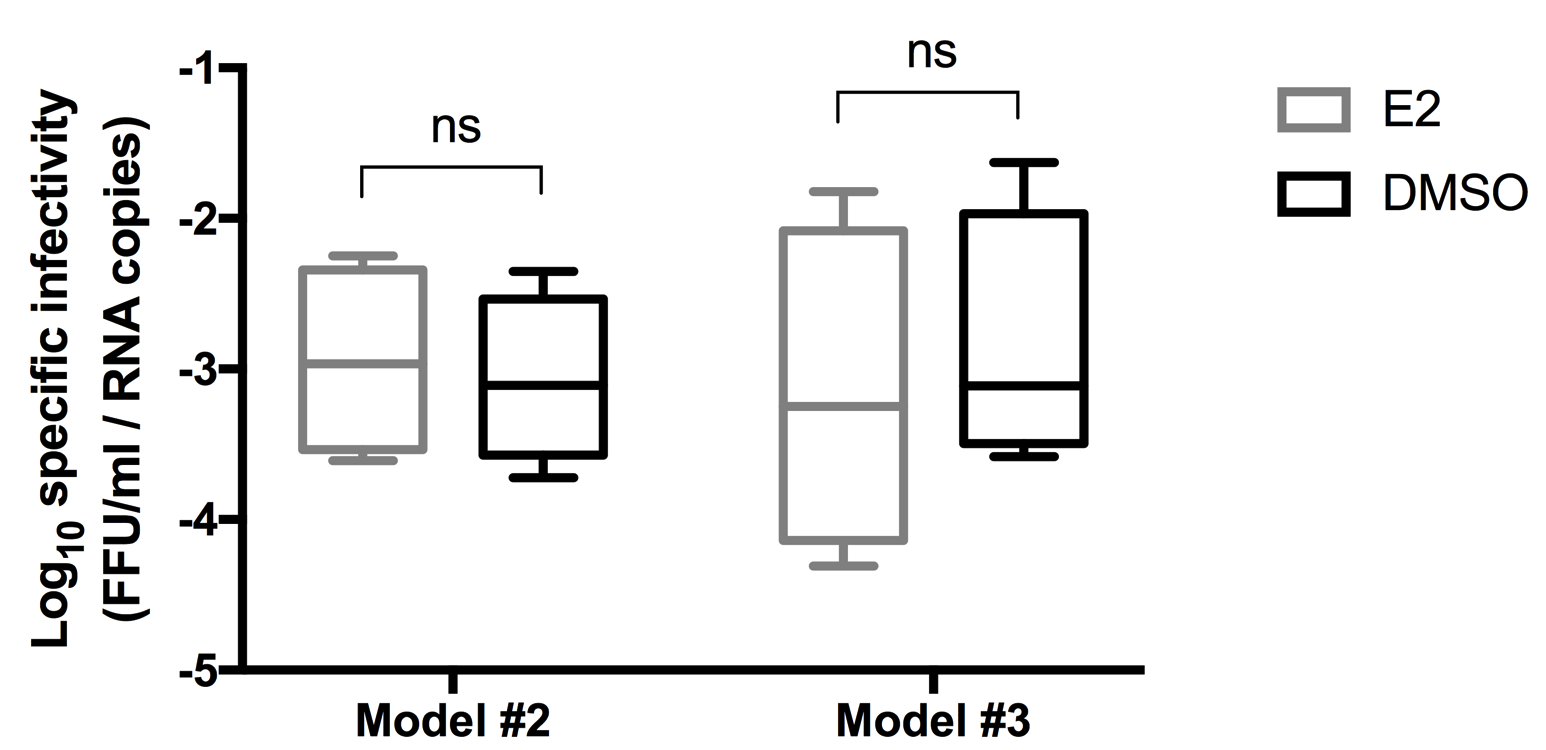

Supplement: Supplementary file 1 [file LIV-37-669-s001.tiff]

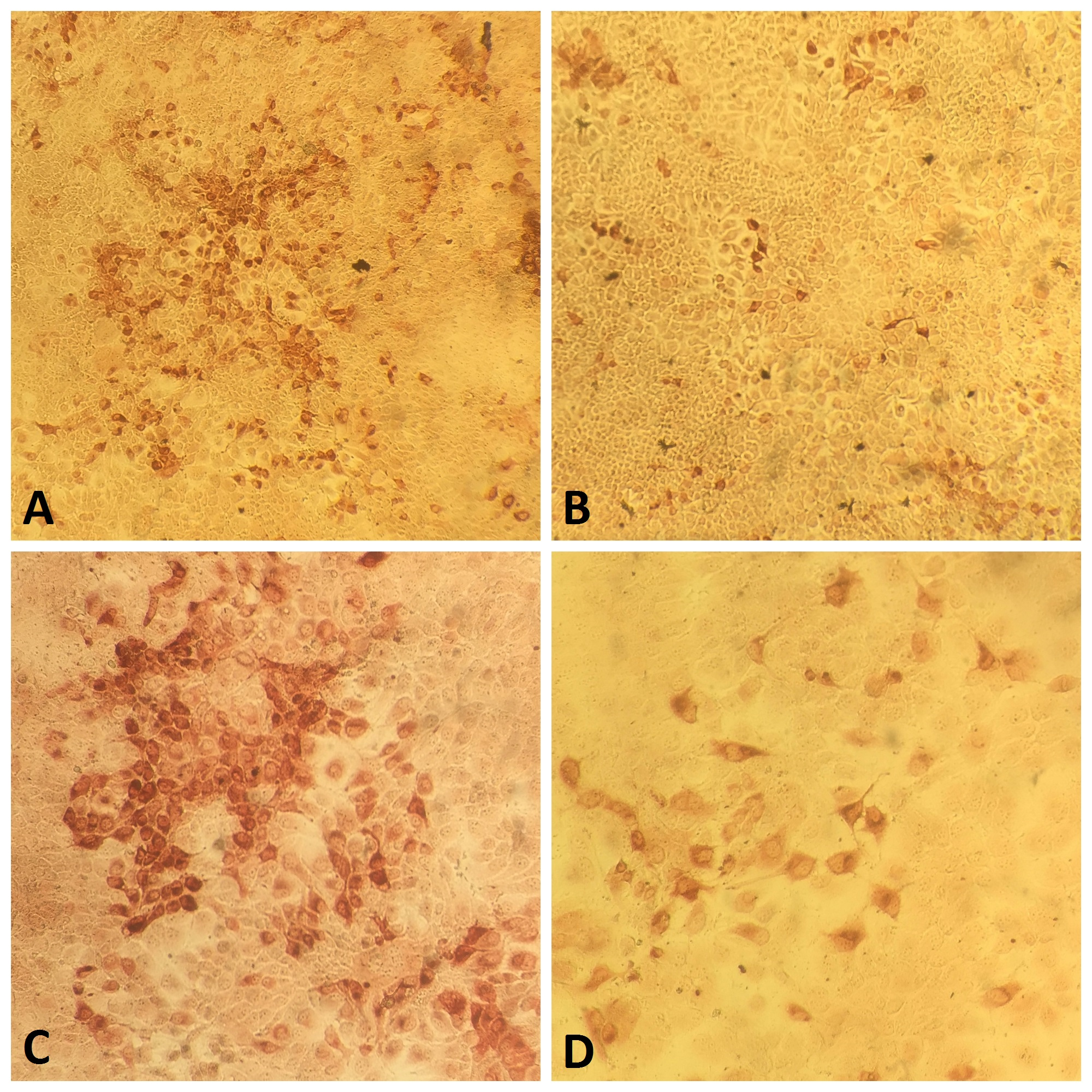

Supplement: Supplementary file 2 [file LIV-37-669-s002.tiff]
