# Supplementary material for: 17,β‐estradiol inhibits hepatitis C virus mainly by interference with the release phase of its life cycle
Source: Liver Int. 2016 Nov 25;37(5):669–77. doi: 10.1111/liv.13303 (PMC5448036; doi:10.1111/liv.13303)
Supplement: Supplementary file 3 [file LIV-37-669-s003.docx]

| **Analyte** |  | **SI Units** |
| --- | --- | --- |
| DHEA-sulfate |  |  |
|  | Male (adult) | 100–6190 μg/L |
|  | Female (adult, premenopausal) | 120–5350 μg/L |
|  | Female (adult, postmenopausal) | 300–2600 μg/L |
| Estradiol |  |  |
|  | Female |  |
|  | Menstruating: |  |
|  | Follicular phase | 74–532 pmol/L |
|  | Midcycle peak | 411–1626 pmol/L |
|  | Luteal phase | 74–885 pmol/L |
|  | Postmenopausal | 217 pmol/L |
|  | Pregnancy: First trimester* | 690-9166 pmol/L |
|  | Pregnancy: Second trimester* | 4691-26402 pmol/L |
|  | Pregnancy: Third trimester* | 22529-12702 pmol/L |
|  | Male | 74 pmol/L |
| Progesterone |  |  |
|  | Female: Follicular | <3.18 nmol/L |
|  | Midluteal | 9.54–63.6 nmol/L |
|  | Male | <3.18 nmol/L |
| Testosterone, total |  |  |
|  | Female | 0.21–2.98 nmol/L |
|  | Male | 9.36–37.10 nmol/L |

Supplementary Table 1: physiological concentrations of sexual hormones in male and female adults, data obtained from Harrison's Principles of Internal Medicine, 19th Edition (1). *data were extrapolated from Abbassi-Ghanavati et al, 2009 (2).

Supplementary figure 1: Specific infectivity of 17, β-estradiol. Huh7 cells were pretreated and infected in the presence of E2 or DMSO according to model #2 and #3. After 3 days the extracellular viral particles were quantitated by RT-qPCR and by titration. Specific infectivity was determined by the ratio of viral titre (expressed as FFU/ml) and the RNA copy number.

Supplementary figure 2: Antiviral effect of 17, β-estradiol . Huh7 cells were infected and treated with E2 following model #2. After 3 days cells were fixed and inhibition was visualised as Focus Forming Units (FFUs) after immune-staining at magnification 4X (A-B) and 10X (C-D) after 72 hr post infection with JFH-1 at MOI 0,1. Figures A and C are DMSO-treated samples, while B-D are E2-stimulated cells (200 nM).

REFERENCES

1. KASPER D L. Harrison's principles of internal medicine. 19th edition / editors, Dennis L. Kasper, MD, William Ellery Channing, Professor of Medicine, Professor of Microbiology, Department of Microbiology and Immunobiology, Harvard Medical School, Division of Infectious Diseases, Brigham and Women's Hospital, Boston, Massachusetts and five others . ed. New York: McGraw Hill Education; 2015.

2. ABBASSI-GHANAVATI M, GREER L G, CUNNINGHAM F G. Pregnancy and laboratory studies: a reference table for clinicians. Obstet Gynecol 2009; 114(6): 1326-31.
